# The Multifactor Measure of Performance: Its Development, Norming, and Validation

**DOI:** 10.3389/fpsyg.2018.00140

**Published:** 2018-02-21

**Authors:** Reuven Bar-On

**Affiliations:** The National Institute for the Regulation of Emergencies and Disasters (NIRED), College of Law and Business, Ramat Gan, Israel

**Keywords:** multifactor measure of performance, MMP3, Reuven Bar-On, performance, assessing and developing performance

## Abstract

This article describes the development as well as the initial norming and validation of the Multifactor Measure of Performance™ (MMP™)^1^, which is a psychometric instrument that is designed to study, assess and enhance key predictors of human performance to help individuals perform at a higher level. It was created by the author, for the purpose of going beyond existing conceptual and psychometric models that often focus on relatively few factors that are purported to assess performance at school, in the workplace and elsewhere. The relative sparsity of multifactorial pre-employment assessment instruments exemplifies, for the author, one of the important reasons for developing the MMP™, which attempts to comprehensively evaluate a wider array of factors that are thought to contribute to performance. In that this situation creates a need in the area of test-construction that should be addressed, the author sought to develop a multifactorial assessment and development instrument that could concomitantly evaluate a combination of physical, cognitive, intra-personal, inter-personal, and motivational factors that significantly contribute to performance. The specific aim of this article is to show why, how and if this could be done as well as to present and discuss the potential importance of the results obtained to date. The findings presented here will hopefully add to what is known about human performance and thus contribute to the professional literature, in addition to contribute to the continued development of the MMP™. The impetus for developing the MMP™ is first explained below, followed by a detailed description of the process involved and the findings obtained; and their potential application is then discussed as well as the possible limitations of the present research and the need for future studies to address them.

## Introduction

While the beta version of Multifactor Measure of Performance™ (referred to as the “MMP1™”), the key predictors of performance it was designed to measure and the initial research involved in developing it were first described by the author in an earlier publication (Bar-On, 2016), the purpose of the present article is to describe the latest version and third revision of the MMP™—“MMP3™”—and how it was created, normed and validated based on research that was conducted since that first publication. This article documents the continuation of the original research that was conducted by the author and first reported in 2016 (Bar-On, 2016) and presents the basic psychometric properties, strengths, and potential applicability of the MMP3™.

The primary purpose and focus of this introductory section is to explain the basic reasons for conducting the initial research that led to the creation of the Multifactor Measure of Performance™ and the specific context in which this work began. One of the author's reasons for beginning this research emerged from reviewing, over the years, a wide variety instruments that were designed to assess various aspects of human behavior and performance. This review indicated, early on, a need for multifactor assessment instruments capable of concomitantly evaluating a combination of predictors of performance, which would hopefully reduce the need for time-consuming and costly *batteries of tests* in psycho-assessment. Additionally, this need to develop a multifactor assessment instrument, designed to measure human performance, emerged from the desire to, metaphorically, “go beyond IQ and EQ” (Bar-On, 2016, p. 104) and include a wide array of physical, cognitive, intra-personal, inter-personal, and motivational contributors to and predictors of performance. Furthermore, the author's overall approach to this endeavor was purposely *a-theoretical* in nature from the outset, in order to avoid being restrained by rigid conceptual frameworks that run the risk of restricting rather than facilitating the ability to examine and potentially include a wider range of contributors to human performance. Essentially, the author envisioned the development of a multifactor assessment instrument that endeavors to include as many significant contributors to performance as possible and to combine them to enhance overall predictive ability.

The above-mentioned need for *a better assessment instrument* was once again confirmed by a survey that the author recently conducted of existing pre-employment tests. Based on a random sample of 120 of the 359 pre-employment tests listed in the 20th edition of the *Mental Measurement Yearbook* (MMY), he found that there appears to be eight major categories describing the vast majority of presently available tests (Carlson et al., 2017). These eight categories are listed in Appendix A and include the number of pre-employment tests identified in each category based on a random sampling of every third test listed in the latest edition of the MMY. A review of Appendix A indicates the percentage of pre-employment tests that are designed to obtain the following information from individuals exploring careers and from job applicants: (1) 9% identify vocational and career interests; (2) 20% evaluate employability as well as general and specific employment skills; (3) 37% examine cognitive or academic potential; (4) 14% assess intra-personal competencies and personality traits; (5) 5% estimate inter-personal compatibility and communication skills; (6) 3% tap managerial and leadership skills; (7) 8% focus on job commitment, social responsibility, work ethics, honesty and/or dependability; and (8) 3% attempt to screen for possible disruptive psychological problems and/or potential criminal behavior. It is interesting to note that almost none of the pre-employment tests reviewed evaluate motivational drive, which is thought to be an important predictor of performance in the workplace and elsewhere (Weitz et al., 1986; Cotton, 1993; Becker et al., 1996; Diefendorff et al., 2002; Locke and Latham, 2002; Halbesleben and Wheeler, 2008; Markos and Sridevi, 2010; Rich et al., 2010). While the MMP3™ was not designed to identify vocational interests or specific occupational skills, it will be shown in the Results section that it is capable of assessing most of the factors that many of the pre-employment tests are designed to evaluate as well as additional factors that they do not assess. The author's examination of the pre-employment tests reviewed by MMY also suggests that they focus on an average of five potential predictors of performance, while the MMP3™ focuses on 25 contributors to performance in the workplace and elsewhere. The MMP3™, moreover, *combines* multifactor contributors to performance in one assessment instrument including (1) physical, (2) cognitive, (3) intra-personal, (4) inter-personal, and (5) motivational factors; and this has the potential of significantly reducing the need to create a battery of pre-employment tests to obtain a more comprehensive evaluation of job applicants, which can be obtained from one assessment instrument (i.e., the MMP3™). Last, the predictive validity of many pre-employment tests listed is not always available in the MMY nor is it frequently convincing when findings are documented in the peer-reviewed literature.

The main reason for developing the Multifactor Measure of Performance™ can be summarized as *the need to develop a better assessment instrument than currently exists*. Moreover, the ultimate aim of creating such an instrument is to substantially contribute to the study, assessment and enhancement of human performance; and the primary purpose of this article is to describe the process involved, which is explained in detail below, as well as to present the key findings, discuss their importance and potential application.

## Methodology

### Sample

The approach used to obtain subjects, in the present study, was to make the 189-item MMP2™ available on SurveyMonkey.com and circulate the link to a number of websites, organizations, and individuals in United States and Canada. This same approach was also used in piloting the beta version of this questionnaire (the MMP1™). In that the initial piloting and norming of this instrument was done on the Internet and not in an academic, medical, or government setting, formal permission was not requested from an official institutional review board to conduct this research. It is also important to convey that the participants were not paid to participate in this research project nor were they coerced to do so in any manner whatsoever. Additionally, it was clearly stated in the introductory section of the MMP2™ that completing the questionnaire was solely for research purposes. In very similar formats moreover, it was also stated at the beginning and at the end of the introductory section that “your agreement to participate in this project, by completing the questionnaire, means that you have given your voluntary consent to do so.”

The above-mentioned process of making the questionnaire available on the Internet generated a sample of, primarily, North American subjects that included a total of 2,380 adults with an average age of 38.0 years. Those who identified their gender and age (*n* = 2,004) included 1,064 males with an average age of 38.4 years and 940 females with an average age of 37.4 years. As is the case with voluntary participation in research, it is difficult to determine the degree to which this population sample represents the total population (although this can be statistically estimated by examining the standard error of the means for the scale scores as is explained in the Results section of this article).

### Data collection

The MMP2™ was the main source of data collection used in the present study to develop of the third and most recent version of the questionnaire—the MMP3™—described in the present article. In an effort to briefly clarify the differences between these three versions of the Multifactor Measure of Performance™, the 216-item “MMP1™” was the beta version, the 189-item “MMP2™” is the second version, and the 142-item “MMP3™” is the third and most recent version. While the development of the MMP1™ was described in an earlier publication in greater detail (Bar-On, 2016), the development the MMP2™ and MMP3™ is described in this article.

The author's approach in developing the original MMP™ was based on the test-construction strategy he applied in developing other psychometric instruments he has developed over the years (Bar-On, 1988, 1997, 2000, 2004, 2006). This involved the following steps: (1) first identifying factors in the literature thought to contribute to performance; (2) receiving input from experienced professionals—“experts”—who have researched, assessed and/or developed performance; (3) then selecting and defining the contributors to and predictors of performance that emerged from his review of the literature and input he received from others; and (4) finally creating, selecting, and further editing scale items. This process created the MMP1™, the beta version of the questionnaire, which comprised 26 primary scales and two validity scales containing a total of 216 items. The two validity scales are unrelated to performance but were included to help examine response accuracy (“Self-Image Accuracy”) and consistency (“Self-Image Consistency”). The Self-Image Accuracy scale originally contained eight items in the MMP1™, while the Self-Image Consistency scale was created by averaging the *absolute differences* in responses to the instrument's highest correlating items across scales. The 26 major scales that were designed to assess contributors to performance, that the author originally identified, and used to develop the MMP1™, are listed in Appendix B. While a detailed description of how the MMP1™ scales were identified, labeled, and defined as well as how the items were selected appear in an earlier publication (Bar-On, 2016), those scales that were retained and included in the MMP3™ are described in the Results section of this article. The response format originally considered was a 5-point Likert scale; however, it was eventually decided to use a slider bar to report responses in percentages based on the author's desire to go from an ordinal to a more statistically sophisticated ratio level of measurement with equal intervals that also include a true zero value (i.e., 0–100%). The MMP1™ was piloted on 997 adults in 2015. The eight items in each scale were then examined with Item Analysis, in order to identify the psychometrically weakest items (i.e., those with the lowest item-scale correlation). This was done to (a) shorten and (b) psychometrically strengthen the questionnaire, by deleting the weakest item and retaining the strongest ones in each scale. This process created the 189-item MMP2™, with 27 scales (including one of the validity scales previously mentioned) comprising seven items in each scale.

An additional source of data collection was the application of a method designed to estimate current occupational performance for those who completed the MMP2™, based on including the following question toward the end of the questionnaire: “If your organization would use the following format to evaluate your work, please indicate how your overall performance was most recently rated on a scale of 0–100%.” The responses from (a) those who answered this question and (b) who also responded with a score of 65% or greater to the following question were used to examine the questionnaire's validity: “Please indicate how often you responded openly and accurately to this questionnaire on a scale of 0–100% of the time.” This generated a sample of 1,788 individuals that was used in examining the questionnaire's discriminatory and predictive validity, which is explained in the data analysis sub-section below.

### Data analysis

In light of the fact that the data collection procedure employed was multivariate in nature, multivariate statistics were applied to examine responses generated by the 189-item MMP2™ and the 142-item MMP3™; and the specific statistical applications applied are described below. The statistical package used by the author was “Statistica 12.7^2^.”

### Exploratory factor analysis (EFA)

EFA was used to estimate the factorial structure of the MMP2™, which guided the development of the MMP3™. The EFA was conducted on the responses generated by the MMP2™ (*n* = 2,380). Nine consecutive EFAs were carried out, in which factor output was limited by progressing from 18 to 26 factors. This was done to estimate the simplest and clearest factorial structure using a varimax normalized rotation. In that the statistical package used did not include oblique rotations, the application of orthogonal rotations needed to be justified and was based on a number of well-documented arguments in the literature since Spearman (1950) and Cattell (1952) first introduced the use of factor analysis in psychology. It has been consistently emphasized that the fundamental goal of EFA is to obtain *a simple factorial structure* that is easy to understand (Cattell, 1978; Yaremko et al., 1986; Bryant and Yarnold, 1995; Kline, 2002; Hill and Lewicki, 2006) and makes good theoretical sense (Kim and Mueller, 1978; Vogt, 1993) irrespective if it was obtained by applying an orthogonal or oblique rotation (Kim and Mueller, 1978; Gorsuch, 1983; Brown, 2009). Moreover, Brown (2009, p. 21) concludes from his review of the literature on EFA that “the choice of rotation may not make much difference.” Kim and Mueller (1978, p. 50) provide one of the most compelling arguments for applying any rotation that generates a simple structure: “Even the issue of whether factors are correlated or not may not make much difference in the exploratory stages of analysis,” and “it even can be argued that employing a method of orthogonal rotation (or maintaining the arbitrary imposition that the factors remain orthogonal) may be preferred over oblique rotation, if for no other reason than that the former is much simpler to understand and interpret.” Hill and Lewicki (2006, p. 238) also focus on this particular issue with oblique rotations that the results obtained are “often not easily interpreted.” Their conclusion stresses the following important point: “If identification of the basic structuring of variables into theoretically meaningful sub-dimensions is the primary concern of the researcher, as is often the case in an exploratory factor analysis, almost any readily available method of rotation will do the job.” Gorsuch (1983, p. 205) also supports this notion and goes one step further: “If the simple structure is clear, any of the more popular procedures can be expected to lead to the same interpretations.” In addition to what many of these and other researchers have found, this important point about rotation selection was empirically demonstrated by Brown (2009, p. 23) who received the identical factorial structure and with very similar factor loadings, by examining the same dataset with three orthogonal rotations and two oblique rotations.

### Basic psychometric properties and reliability

Based on the results of EFA, that guided the continued development of the Multifactor Measure of Performance™, the basic psychometric properties of the MMP3™ were examined and are presented in the Results section together with a description of the questionnaire's reliability. This was also conducted on the responses to the questionnaire generated by the North American normative sample (*n* = 2,380).

### Analysis of variance (ANOVA)

ANOVA was applied to evaluate MMP3™'s discriminatory validity, by examining the differences between high and low performers. “High performers” were those who scored +1 SD above the mean on self-reported performance (*n* = 304), as described in the Methodology section, while “low performers” were those who scored −1 SD below the mean (*n* = 292) for this estimate of performance. ANOVA was conducted on the responses to the questionnaire generated by those individuals, in the normative sample, who provided a self-reported estimate of their current occupational performance as well as a score 65% or higher on accurate and honest responding as was previously described.

### Multiple regression analysis (MRA)

MRA was used to examine MMP3™'s predictive validity. The MRA was also conducted on the responses to the questionnaire generated by those individuals who provided a self-reported estimate of their current occupational performance as well as scored 65% or higher on accurate and honest responding (*n* = 1,788). A forward stepwise analysis was applied.

## Results

### MMP3™'s factorial structure

The simplest and most logical factorial structure of the MMP3™ emerged from limiting factor output to 22 factors, based on conducting nine consecutive EFAs in examining the MMP2™'s responses obtained from 2,380 adults who completed the questionnaire. The results are displayed in Tables 1A–C.

**Table 1:** The scale items' highest loadings on the clearest defined factors and sub-factors that emerged from EFA (*n* = 2,380), appearing in parenthesis following the number of each of these items as listed in the MMP™2.

**A**				
**F1**	**F2**	**F3**	**F4**	**F6**
**Engagement**	**General Cognitive Competence**	**Coping and Endurance**	**Connectedness**	**Self-Reliance**
19. (0.59)	3. (0.53)	44. (0.56)	24. (0.53)	14. (0.54)
46. (0.67)	30. (0.62)	71. (0.54)	78. (0.57)	41. (0.72)
73. (0.49)	57. (0.57)	98. (0.58)	105. (0.61)	68. (0.76)
100. (0.76)	84. (0.51)	125. (0.69)	132. (0.56)	95. (0.63)
127. (0.66)	111. (0.45)	152. (0.64)	159. (0.59)	149. (0.51)
154. (0.59)		Situational Awareness	186. (0.37)	
181. (0.62)		4. (0.33)		
		58. (0.40)		
		139. (0.47)		
		166. (0.43)		
		Flexibility		
		60. (0.56)		
		87. (0.58)		
		114. (0.58)		
		141. (0.43)		
		168. (0.37)		
		Resourcefulness and Resilience		
		61. (0.51)		
		88. (0.56)		
		115. (0.52)		
		142. (0.58)		
		Decision-Making		
		62. (0.58)		
		89. (0.51)		
		116. (0.61)		
		143. (0.61)		
		170. (0.63)		
		Preparedness and Readiness		
		63. (0.57)		
		64. (0.54)		
		91. (0.60)		
		144. (0.52)		
		171. (0.58)		
Var. = 7.04%	Var. = 5.82%	Var. = 20.75%	Var. = 5.48%	Var. = 3.08%
**B**				
**F7**	**F8**	**F9**	**F11**	
**Physical Fitness and Well-Being**	**Courage**	**Discomfort Tolerance and Stamina**	**Decisiveness**	
1. (0.71)	15. (0.63)	83. (0.65)	13. (0.49)	
28. (0.83)	42. (0.69)	110. (0.64)	40. (0.60)	
54. (0.81)	69. (0.53)	137. (0.76)	67. (0.60)	
55. (0.56)	96. (0.54)	164. (0.65)	94. (0.50)	
81. (0.71)	177. (0.42)		175. (0.41)	
109. (0.46)				
163. (0.38)				
Var. = 6.37%	Var. = 3.40%	Var. = 2.72%	Var. = 3.31%	
**C**				
**F12**	**F14**	**F15**	**F19**	**F20**
**Applying Experience**	**Social Awareness**	**Self-Motivation**	**Self-Control**	**Meaningfulness**
5. (0.48)	23. (0.62)	18. (0.46)	16. (0.61)	26. (0.51)
32. (0.50)	50. (0.74)	45. (0.28)	43. (0.53)	80. (0.55)
59. (0.49)	77. (0.77)	72. (0.53)	70. (0.55)	107. (0.40)
86. (0.65)	104. (0.47)	99. (0.30)	97. (0.54)	134. (0.47)
113. (0.70)		Determination	124. (0.53)	161. (0.36)
140. (0.59)		20. (0.50)	178. (0.44)	Social Responsibility
167. (0.57)		47. (0.68)		25. (0.38)
		74. (0.48)		52. (0.31)
		128. (0.56)		79. (0.43)
		155. (0.68)		133. (0.27)
		182. (0.53)		
		Perseverance		
		21. (0.48)		
		48. (0.56)		
		75. (0.70)		
		102. (0.44)		
		156. (0.50)		
Var. = 4.60%	Var. = 3.76%	Var. = 8.10%	Var. = 4.07%	Var. = 3.27%

In addition to being logical and relatively uncomplicated to interpret, the results appearing in Tables 1A–C suggest that the estimated factorial structure of the MMP3™ accounts for nearly 82% of the total variance that it was designed to capture. Additionally, by comparing the original MMP1™ scales with those that were retained in the MMP3™, as shown in Appendix B, ~85% of them were retained. The results indicate that 14 factors and 11 sub-factors^3^ emerged from EFA, with two or more of them loading on factors 3, 15, and 20. In accordance with requiring *most* of the highest loading items from the original scales being examined load on the resultant output factors that emerge, as suggested by Cattell (1952, 1978), Anastasi (1988), Tabachnick and Fidell (2001), a minimum of 4 out of the original 7 MMP2™ scale items was used in identifying the factors in Tables 1A–C. This approach guided the development of the MMP3™ with its 14 scales and 11 sub-scales^4^ that were labeled according to the names of the original MMP1™/MMP2™ scales as well as the specific nature of the highest loading items. As a result of this approach, the labels of a few of the original MMP1™ scales were mildly altered. The MMP3™ scales currently comprise a minimum of 7 items, while the sub-scales contain a minimum of 5 items; and here it is important to mention that 5 items per scale is justified if there is an inner-correlation between them that is equal to or greater than 0.70 (Drolet and Morrison, 2001; Worthigton and Whittaker, 2006; Bergkvist and Rossiters, 2007; Hair et al., 2011), which was confirmed by the results in Table 3.

The results in Table 1A suggest that an additional sub-factor has apparently emerged from EFA, which was not previously considered or described by the author. The highest loading items on this new sub-factor originated from MMP1™'s (a) General Cognitive Competence, (b) Action-Planning, (c) Rapid Implementation, and (d) Discomfort Tolerance scales. An examination of the highest loading items, moreover, suggests that this new sub-factor appears to be associated with being prepared and ready to cope with situations requiring rapid execution of some immediate form of goal-oriented action. It was, therefore, decided to add “Preparedness and Readiness” as an additional MMP3™ sub-scale designed to assess this particular sub-factor.

A further examination of the factor loadings in Tables 1A–C reveals an average factor loading of 0.55, which could suggest the potential for significant factorial validity; however, this will need to be confirmed by Confirmatory Factor Analysis (CFA) in future studies that will need to be conducted on larger and more diverse independent samples. Additionally, only four out of the 113 highest loadings are lower than 0.32 with the lowest being 0.27. While 0.30 has been considered the minimum loading for inclusion in resultant factors (Tabachnick and Fidell, 2001), some consider loadings even lower than 0.30 to be acceptable for samples larger than 100 (Kline, 2002, p. 52–53).

The MMP3™ presently contains 142 items loading on 14 scales and 11 sub-scales, which takes an average of 25 min to complete based on a North American sample of 468 adults who recently completed this version of the questionnaire.

When the order of the resultant factors in Tables 1A–C are rearranged in a theoretically logical order, the following would appear to be the structure of the factors and sub-factors that are assessed with the MMP3™ scales and sub-scales (which will need to be confirmed by CFA to verify this apparent structure):

1. Physical Fitness and Well-Being
2. Discomfort Tolerance and Stamina
3. General Cognitive Competence
4. Key Cognitive Competencies
  4.1 Coping and Endurance
  4.2 Situational Awareness
  4.3 Flexibility
  4.4 Resourcefulness and Resilience
  4.5 Decision-Making
  4.6 Preparedness and Readiness
5. Applying Experience
6. Self-Control
7. Self-Reliance
8. Decisiveness
9. Courage
10. Social Awareness
11. Connectedness
12. Finding Meaning and Acting Responsibly
  12.1 Meaningfulness
  12.2 Social Responsibility
13. Engagement
14. Motivational Drive
  14.1 Self-Motivation
  14.2 Determination
  14.3 Perseverance

According to how the above factors were originally defined by the author (Bar-On, 2016) and based on the MMP2™ items that loaded the highest on the factors and sub-factors that emerged from EFA listed in Tables 1A–C, the MMP3™ scales and sub-scales are thought to assess the following contributors to performance:

- **Physical Fitness and Well-Being:** In addition to “striving to obtain and maintain good physical fitness” (Bar-On, 2016, p. 104) which contributes to “overall well-being” (Bar-On, 2016, p. 106), this scale emerged from EFA as a combination of two MMP2™ scales which impacts performance. It appears to assess how people feel in general about their physical fitness, eating, and sleeping habits as well as how energetic they typically are in what they do.
- **Discomfort Tolerance and Stamina:** In addition to the ability to “temporarily suspend everyday physical needs and comforts in order to complete a task” (Bar-On, 2016, p. 104), this scale assesses the willingness to eat at irregular times, work longer hours with less sleep as well as to work on weekends in order to meet deadlines and finish the work on time. It also appears to measure stamina and the ability to continue functioning when need be.
- **General Cognitive Competence:** In addition to one's ability to “learn new information and apply learned knowledge, logic, and reasoning for the purpose of understanding and solving problems” (Bar-On, 2016, p. 105), this scale appears to assess the capacity to learn more about the challenges one is faced with, to first understand them and then think about a reasonable course of action, to apply potentially effective solutions and weigh conflicting evidence, as well as to take into account the short-term and long-term consequences of potential solutions being considered.
- **Key Cognitive Competencies:** This composite scale^5^ was created to measure a factor that emerged from EFA, which appears to collectively assesses the following six sub-factors that are important contributors to effective cognitive functioning:
  **◦ Coping and Endurance:** In addition to “managing one's feelings in stressful situations” in order to function effectively while remaining relatively calm (Bar-On, 2016, p. 106), this sub-scale appears to assess how well people typically cope and function under pressure. This includes evaluating how effective they are in dealing with anxiety-provoking situations.
  **◦ Situational Awareness:** In addition to “evaluating the immediate situation, paying attention to detail as well as understanding, clarifying, and closing gaps between the perception of subjective reality and objective reality” (Bar-On, 2016, p. 105), this sub-scale assesses how attentive people are to their surroundings and how well they size up the situation. This appears to be based on an ability to update their assessment of situations in response to changes in the immediate environment as well as to filter out irrelevant information, in order not to get distracted.
  **◦ Flexibility:** In addition to “coping with and adapting to change as well as dealing with unexpected, unpredictable and confusing situations” (Bar-On, 2016, p. 105), this sub-scale assesses the ability to flexibly “think on one's feet” and deal with the unexpected, finding ways to improvise and adapt when the unpredictable occurs, and to make the necessary adjustments to overcome. This often requires one to re-reframe setbacks and not to see them as personal or permanent.
  **◦ Resourcefulness and Resilience:** In addition to the ability “to be innovative and consider different ways of coping with situations” (Bar-On, 2016, p. 105), this sub-scale appears to assess the capacity of individuals to generate different approaches to dealing with challenges and setbacks as well as to resiliently recover from them. If previous approaches are ineffective, resourceful individuals typically come up with alternative approaches that work; and this often depends on formulating an effective course of action aimed at going from the current situation to a better one.
  **◦ Decision-Making:** In addition to “generating potentially effective solutions to problems, weighing the pros and cons of each possibility and deciding on the best solution” (Bar-On, 2016, p. 105), this sub-scale assesses the ability to make good decisions in general. Moreover, this scale measures the ability to come up with a potentially effective plan that requires coping with ambiguity and exercising sound judgement even when working under pressure and dealing with potential risks.
  **◦ Preparedness and Readiness:** This sub-scale was created to measure a sub-factor that surfaced as a result of EFA. Based on the items that loaded on this sub-factor, this new sub-scale appears to assess the ability of individuals to be prepared and ready to cope with immediate situations that arise and/or to execute some form of goal-oriented action based on what they have learned. This includes immediately sizing up what is presently happening in the here-and-now, deciding on the best course of action and rapidly implementing it, which appears to be what is cognitively needed in dealing with emergency situations.
- **Applying Experience:** In addition to “appropriately and effectively applying past experience in order to facilitate current problem-analysis, problem-solving, and decision-making” (Bar-On, 2016, p. 105), this scale assesses the ability to apply experience in understanding and dealing with current challenges and problematic situations. This requires the capacity to effectively combine past experience with new information and approaches, which is an important contributor to effective cognitive functioning.
- **Self-Control:** In addition to “controlling emotions and maintaining self-composure” (Bar-On, 2016, p. 106), this scale essentially assesses “the ability of people to control their emotions so they work for them and not against them.” Moreover, it evaluates the ability to effectively deal with challenges while maintaining outward composure.
- **Self-Reliance:** In addition to “being independent from others and being able to think things out alone, make decisions and act independently when needed” (Bar-On, 2016, p. 105), this scale essentially assesses the capacity to think and act independently rather than depending on others. It evaluates the ability of people to act alone when need be, even though they are open to receiving input and suggestions from others.
- **Decisiveness:** In addition to “expressing oneself openly, clearly and boldly” as well as “being able to confidently convey feelings, beliefs, and ideas” (Bar-On, 2016, p. 105), this scale assesses the ability of people to be assertive and decisive as well as to set firm limits with others when necessary but without being aggressive or hostile.
- **Courage:** In addition to being able “to overcome one's apprehensions and fears to act courageously” (Bar-On, 2016, p. 105–106), this scale was designed to measure the capability of individuals to protect, stand up for and support others even when there might be negative consequences for doing so. Additionally, this scale also evaluates the extent to which people are even prepared to risk their life to save another person's life.
- **Social Awareness:** In addition to “being aware of others, their feelings and concerns which helps one interact with people and become a more cooperative, constructive, and contributing team player” (Bar-On, 2016, p. 106), this scale assesses the ability to understand non-verbal communication, to know how others feel and to be attentive to their needs.
- **Connectedness:** In addition to “being able to connect with other people and to establish and maintain mutually satisfying interpersonal relationships” (Bar-On, 2016, p. 106), this scale appears to evaluate the capacity to establish and maintain good relationships with others, get along with friends and colleagues as well as to enjoy social interactions in general.
- **Finding Meaning and Acting Responsibly:** This is a composite scale that was created to measure a factor that emerged from EFA, that assesses the following two sub-factors that are thought to be important contributors to finding meaning in what one does which also benefits others as well as oneself in a socially responsible manner:
  **◦ Meaningfulness:** In addition to “finding meaning in what one does as well as being passionately involved in meaningful pursuits that benefit others in addition to oneself” (Bar-On, 2016, p. 106), this sub-scale appears to assess the ability to live a meaningful and rewarding life.
  **◦ Social Responsibility:** In addition to “living according to a set of principles, values, and beliefs which guide one's decisions and ability to do the right thing” (Bar-On, 2016, p. 106), this sub-scale appears to assess the consistency of one's moral integrity when one is with family members, friends and/or colleagues. Fundamentally, this is based on the ability to understand the difference between *right* and *wrong* and to act accordingly.
- **Engagement:** In addition to “being committed to one's work which builds on feeling passionate about what one enjoys doing” that enhances overall motivational drive (Bar-On, 2016, p. 106), this scale assesses the degree to which people feel positive about what they do or have done and understand the positive impact it has or might have on others.
- **Motivational Drive:** This is a composite scale that was created to measure a factor that emerged from EFA, which collectively assesses the following three sub-factors that are thought to be important contributors to one's overall motivational drive that significantly impacts performance:
  **◦ Self-Motivation:** In addition to “being positive, optimistic, and energized in doing what one does” (Bar-On, 2016, p. 106), this sub-scale assesses the degree to which people are capable of motivating themselves. This enhances their drive to get as much as they can out of what they enjoy doing and energizes them to perform on an even higher level.
  **◦ Determination:** In addition to “being committed to decisions that are made and goals that are set as well as being determined to follow through with them” (Bar-On, 2016, p. 106), this sub-scale appears to assess how resolute people are in the choices and decisions they make. This essentially requires the resolve to begin what they decide to do and to move into action mode after decisions are made.
  **◦ Perseverance:** In addition to “persevering and following through with a task until it is completed” (Bar-On, 2016, p. 106), this sub-scale also assesses the drive to pursue goals in general.

### MMP3™'s basic psychometric properties

Subsequent to obtaining an estimate of MMP3™'s factorial structure and what it appears to assess based on the highest loading items, the basic psychometric properties of this developing questionnaire were then examined by evaluating the scale score means, standard deviations, standard error of means, skewness, and kurtosis. The results are presented in Table 2A. Mean scores for males and females as well as for five different age groups were also examined for significant differences, and the results are presented in Tables 2B,C respectively. It is important to note that mean scores appear in percentages, in that the response options were formatted in percentages ranging from 0 to 100% as was previously described in the Methodology section. To reiterate moreover, the term “composite score” is descriptively used to refer to the following three scale scores that were created by averaging the sub-scales that they comprise: (1) Key Cognitive Competencies; (2) Finding Meaning and Acting Responsibly; and (3) Motivational Drive.

**Table 2A:** The basic psychometric properties of the MMP3™, including mean scores and standard deviations (*SD*) in percentages, standard error of means (SEM), skewness (Skew.), and kurtosis (Kurt.), based on the normative sample (*n* = 2,380).

**MMP3™ scales**	**Mean (%)**	***SD* (%)**	**SEM**	**Skew**.	**Kurt**.
1. Physical Fitness and Well-Being	68.1	17.7	0.51	−0.44	−0.05
2. Discomfort Tolerance and Stamina	71.8	14.5	0.42	−0.48	0.11
3. General Cognitive Competence	76.8	13.3	0.38	−0.58	0.68
4. Key Cognitive Competencies	73.9	13.5	0.39	−0.60	1.00
4.1 Coping and Endurance	72.9	17.3	0.50	−0.74	0.82
4.2 Situational Awareness	72.9	14.1	0.41	−0.52	0.74
4.3 Flexibility	74.0	15.1	0.44	−0.68	1.26
4.4 Resourcefulness and Resilience	74.0	14.9	0.43	−0.37	0.12
4.5 Decision-Making	74.0	14.9	0.43	−0.45	0.49
4.6 Preparedness and Readiness	75.4	15.5	0.45	−0.65	0.45
5. Applying Experience	80.2	13.9	0.40	−0.79	1.23
6. Self-Control	73.0	15.2	0.44	−0.52	0.37
7. Self-Reliance	70.9	13.6	0.39	−0.45	0.54
8. Decisiveness	67.3	15.3	0.44	−0.51	0.35
9. Courage	68.8	15.6	0.45	−0.23	−0.38
10. Social Awareness	72.2	13.9	0.40	−0.46	0.51
11. Connectedness	73.4	14.6	0.42	−0.70	1.23
12. Finding Meaning and Acting Responsibly	82.4	12.2	0.35	−0.95	1.57
12.1 Meaningfulness	80.6	14.3	0.42	−0.84	1.01
12.2 Social Responsibility	84.2	12.7	0.37	−1.05	1.64
13. Engagement	76.3	17.2	0.50	−0.87	0.75
14. Motivational Drive	79.2	13.7	0.40	−0.82	0.93
14.1 Self-Motivation	79.2	14.6	0.42	−0.88	1.19
14.2 Determination	78.8	15.0	0.43	−0.71	0.42
14.3 Perseverance	79.6	14.6	0.42	−0.79	0.61

**Table 2B:** The MMP3™ scale scores, in percentages, for males (*n* = 1,064) and females (*n* = 940), based on individuals who identified their gender when responding to the questionnaire.

**MMP3™ Scales**	**Males (%)**	**Females (%)**	***F*-value**	***p*-level**
1. Physical Fitness and Well-Being	71.1	66.0	18.98	<0.001
2. Discomfort Tolerance and Stamina	72.8	70.9	4.41	0.036
3. General Cognitive Competence	77.2	76.3	1.71	0.192
4. Key Cognitive Competencies	75.2	72.6	10.11	0.002
4.1 Coping and Endurance	74.9	70.8	15.07	<0.001
4.2 Situational Awareness	74.0	71.5	9.17	0.003
4.3 Flexibility	74.6	73.1	2.72	0.099
4.4 Resourcefulness and Resilience	75.4	73.0	6.92	0.009
4.5 Decision-Making	75.4	72.6	10.41	0.001
4.6 Preparedness and Readiness	76.7	74.8	4.67	0.031
5. Applying Experience	80.9	79.5	2.01	0.157
6. Self-Control	74.6	71.6	12.16	0.001
7. Self-Reliance	69.8	71.8	5.72	0.017
8. Decisiveness	68.7	65.5	10.76	0.001
9. Courage	71.7	65.6	41.32	<0.001
10. Social Awareness	70.7	74.3	13.22	<0.001
11. Connectedness	73.3	74.5	1.20	0.273
12. Finding Meaning and Acting Responsibly	82.0	82.8	0.99	0.321
12.1 Meaningfulness	80.3	80.9	0.63	0.429
12.2 Social Responsibility	83.8	84.6	1.03	0.311
13. Engagement	78.3	75.2	6.46	0.011
14. Motivational Drive	80.2	78.8	1.43	0.233
14.1 Self-Motivation	80.4	78.8	2.27	0.132
14.2 Determination	79.8	78.4	1.19	0.276
14.3 Perseverance	80.4	79.4	0.53	0.468

*ANOVA was used to examine potential gender differences*.

**Table 2C:** The MMP3™ scale scores, in percentages, for five different age groups [18–29 (*n* = 712), 30–39 (*n* = 326), 40–49 (*n* = 400), 50–59 (*n* = 334) and ≥60 (*n* = 138)] based on individuals who identified their age when responding to the questionnaire.

**MMP3™ scales**	**18–29 (%)**	**30–39 (%)**	**40–49(%)**	**50–59(%)**	**≥60(%)**	***F*-value**	***p*-level**
1. Physical Fitness and Well-Being	72.5	66.3	63.7	67.9	72.2	9.64	<0.001
2. Discomfort Tolerance and Stamina	72.1	70.5	71.3	72.1	75.6	1.47	0.211
3. General Cognitive Competence	75.5	77.1	76.2	79.6	79.3	3.57	0.007
4. Key Cognitive Competencies	72.7	73.3	74.0	76.8	76.7	3.51	0.007
4.1 Coping and Endurance	71.1	71.9	73.7	76.3	77.0	3.65	0.006
4.2 Situational Awareness	72.5	71.1	72.8	74.9	76.2	2.29	0.058
4.3 Flexibility	71.6	73.5	75.0	77.4	76.1	5.03	0.001
4.4 Resourcefulness and Resilience	73.0	74.2	73.0	77.9	75.8	3.69	0.005
4.5 Decision-Making	72.4	73.7	73.9	77.1	77.0	3.45	0.008
4.6 Preparedness and Readiness	75.5	75.1	75.4	77.5	78.2	1.00	0.408
5. Applying Experience	78.5	80.6	80.9	82.5	82.5	2.93	0.020
6. Self-Control	73.3	70.7	73.0	74.8	76.2	2.10	0.079
7. Self-Reliance	67.7	70.5	72.5	72.1	74.7	7.05	<0.001
8. Decisiveness	66.5	66.0	66.9	69.5	67.7	1.29	0.273
9. Courage	66.6	66.2	70.1	73.0	73.6	8.25	<0.001
10. Social Awareness	69.8	73.5	73.7	74.6	72.6	4.70	0.001
11. Connectedness	75.1	71.7	72.9	74.9	74.5	2.09	0.080
12. Finding Meaning and Acting Responsibly	79.6	81.5	84.1	86.2	86.0	11.96	<0.001
12.1 Meaningfulness	78.9	79.0	81.7	83.7	83.8	4.87	0.001
12.2 Social Responsibility	80.3	84.0	86.5	88.8	88.2	18.42	<0.001
13. Engagement	77.7	75.0	73.3	79.5	81.4	4.92	0.001
14. Motivational Drive	81.2	77.4	77.3	79.8	83.2	4.84	0.001
14.1 Self-Motivation	82.2	77.3	76.5	79.8	81.5	6.58	<0.001
14.2 Determination	80.0	77.0	77.4	80.0	83.5	3.29	0.011
14.3 Perseverance	81.4	77.9	78.0	79.7	84.5	4.12	0.003

*ANOVA was used to examine potential age differences*.

In that the skewness and kurtosis of all of the composite scales, scales and sub-scales fall within the acceptable ±2.0 range (Trochim and Donnelly, 2014), the mean scores in Table 2A appear to be normally distributed. Moreover, the standard error of the means obtained for the scale scores suggests it is expected that similar results would be obtained from other samples from the same population (Tabachnick and Fidell, 2001; Barde and Prajakt, 2012). The mean scores, however, appear to be relatively high, substantially above an expected middle range, which suggests a need to adjust raw scores proportionally downward. Subsequent to collecting data from larger and more diverse samples across cultures, this will eventually be done by multiplying raw scores by non-standardized beta weights obtained from applying MRA in order to first examine the degree of correlation between the major scale scores and the validity scale score that attempts to assess social response bias. The software, used to score the responses, will then be programmed to automatically and proportionally reduce significantly high raw scores by converting them to adjusted scores thus improving the accuracy of the scores (i.e., raw scores adjusted through *factoring out* response bias).

While no significant differences were found in (a) general cognitive competence, (b) the ability to apply experience in coping with problems, (c) connecting with others, (d) finding meaning in one's work and acting responsibly, or in (e) motivational drive, significant gender differences are found in more than half of the scale scores appearing in Table 2B; and the majority of these scores were apparently higher for males in the North American samples examined. As such, gender-specific norms will be used in the future to score MMP3™ responses in order to generate more accurate results (i.e., raw scores adjusted by *factoring out* gender interaction).

The results in Table 2C indicate that most contributors to performance tend to increase with age, which suggests that older people perform better than younger people in general. While this might be the results of *experience-based wisdom*, this will need to be empirically examined in future studies. In any event, age-specific norms will be used in scoring MMP3™ responses in addition to gender-specific norms as was previously mentioned.

### MMP3™'s reliability

The primary approach used to estimate MMP3™'s reliability was to evaluate the internal consistency of its scales with Cronbach alphas; and the results are presented in Table 3.

**Table 3:** The internal consistency reliability of the MMP3™ scales, based on the normative sample (*n* = 2,380).

**MMP3™ scales**	**Cronbach alphas**
1. Physical Fitness and Well-Being	0.86
2. Discomfort Tolerance and Stamina	0.73
3. General Cognitive Competence	0.80
4. Key Cognitive Competencies	0.96
4.1 Coping and Endurance	0.87
4.2 Situational Awareness	0.73
4.3 Flexibility	0.78
4.4 Resourcefulness and Resilience	0.80
4.5 Decision-Making	0.84
4.6 Preparedness and Readiness	0.82
5. Applying Experience	0.86
6. Self-Control	0.87
7. Self-Reliance	0.72
8. Decisiveness	0.81
9. Courage	0.77
10. Social Awareness	0.79
11. Connectedness	0.82
12. Finding Meaning and Acting Responsibly	0.84
12.1 Meaningfulness	0.79
12.2 Socially Responsible	0.74
13. Engagement	0.88
14. Motivational Drive	0.93
14.1 Self-Motivation	0.78
14.2 Determination	0.85
14.3 Perseverance	0.79

The results in Table 3 suggest that all of the scales possess more than adequate reliability, based on the assumption that reliability coefficients equal to or greater than 0.70 indicate adequate reliability for scales and sub-scales (Anastasi, 1988; Tabachnick and Fidell, 2001) while coefficients equal to or greater than 0.80 are thought to be the minimum for composite scales which has been achieved in this study. These findings also justify the creation of sub-scales comprising as few as five items, as was previously mentioned (Hair et al., 2011). Furthermore, relatively high reliability coefficients usually predict that the scale's validity will be relatively high as well (Hill and Lewicki, 2006).

### MMP3™'s validity

Discriminant validity and predictive validity were examined with ANOVA and MRA respectively. In the ANOVA evaluation of MMP3™'s discriminant validity, a sample of high performers and low performers were compared for possible significant differences based on their self-reported performance ratings at work. High performers were those whose recent performance rating was self-reported to be equal to or greater than one standard deviation above the mean (*n* = 304), while low performers were those whose performance rating was equal to or less than one standard deviation from the mean (*n* = 292) as was previously explained in the Methodology section. Although “self-reported performance ratings” are most likely biased as explained in that section, the method applied to calculate them may have provided a potentially accurate estimate of current occupational performance (which will need to be examined by more objective methods in future studies). The initial results for discriminatory validity are listed in Table 4. Additionally, predictive validity was evaluated by applying MRA to examine the ability of the MMP3™ scores to predict self-reported performance (*n* = 1,788); and the results are presented in Table 5.

**Table 4:** The ability of the MMP3™ to distinguish between high performers (*n* = 304) and low performers (*n* = 292), examined by applying ANOVA with gender and age designated as co-variates.

**MMP3™ scales**	**High**	**Low**	***F*-value**	***p*-level**
	**(%)**	**(%)**		
1. Physical Fitness and Well-Being	77.0	63.9	22.18	<0.001
2. Discomfort Tolerance and Stamina	75.9	67.7	12.65	0.005
3. General Cognitive Competence	82.0	70.3	38.68	<0.001
4. Key Cognitive Competencies	80.7	65.6	56.75	<0.001
4.1 Coping and Endurance	80.6	61.9	47.62	<0.001
4.2 Situational Awareness	80.0	64.1	59.15	<0.001
4.3 Flexibility	78.3	66.4	27.78	<0.001
4.4 Resourcefulness and Resilience	80.6	67.4	35.56	<0.001
4.5 Decision-Making	81.3	66.3	50.05	<0.001
4.6 Preparedness and Readiness	83.7	67.7	50.26	<0.001
5. Applying Experience	82.1	76.6	10.32	0.003
6. Self-Control	79.7	65.2	39.94	<0.001
7. Self-Reliance	69.9	71.5	0.69	0.408
8. Decisiveness	72.0	62.2	19.67	<0.001
9. Courage	71.8	62.2	22.81	<0.001
10. Social Awareness	75.1	67.5	18.02	<0.001
11. Connectedness	79.0	67.4	28.78	<0.001
12. Finding Meaning and Acting Responsibly	86.7	77.1	33.55	<0.001
12.1 Meaningfulness	85.0	75.3	24.99	<0.001
12.2 Social Responsibility	88.3	78.8	30.49	<0.001
13. Engagement	83.5	69.4	33.66	<0.001
14. Motivational Drive	86.1	73.1	41.61	<0.001
14.1 Self-Motivation	86.4	72.4	41.76	<0.001
14.2 Determination	85.6	73.8	28.49	<0.001
14.3 Perseverance	86.3	73.3	36.78	<0.001

**Table 5:** The ability of the MMP3™ to predict performance, based on applying MRA to examine the degree of correlation between its scale scores and self-reported performance at work (*n* = 1,788).

**MMP3™ scales**	**Multiple R**	***F*-value**	***p*-level**
1. Physical Fitness and Well-Being	0.23	18.78	<0.001
2. Discomfort Tolerance and Stamina	0.26	36.97	<0.001
3. General Cognitive Competence	0.33	40.64	<0.001
4. Key Cognitive Competencies	0.43	18.64	<0.001
4.1 Coping and Endurance	0.35	47.78	<0.001
4.2 Situational Awareness	0.37	39.27	<0.001
4.3 Flexibility	0.29	24.02	<0.001
4.4 Resourcefulness and Resilience	0.25	16.74	<0.001
4.5 Decision-Making	0.32	28.20	<0.001
4.6 Preparedness and Readiness	0.31	21.63	<0.001
5. Applying Experience	0.23	11.23	<0.001
6. Self-Control	0.31	18.38	<0.001
7. Self-Reliance	0.25	16.84	<0.001
8. Decisiveness	0.21	22.81	<0.001
9. Courage	0.26	14.91	<0.001
10. Social Awareness	0.22	10.53	<0.001
11. Connectedness	0.30	14.21	<0.001
12. Finding Meaning and Acting Responsibly	0.33	15.81	<0.001
12.1 Meaningfulness	0.26	17.82	<0.001
12.2 Social Responsibility	0.29	31.54	<0.001
13. Engagement	0.26	18.36	<0.001
14. Motivational Drive	0.32	16.74	<0.001
14.1 Self-Motivation	0.29	22.80	<0.001
14.2 Determination	0.24	21.35	<0.001
14.3 Perseverance	0.29	46.31	<0.001

The findings in Table 4 indicate that nearly all of the MMP3™ scales are capable of significantly discriminating between high and low performers in the present study, while the only scale that could not significantly discriminate between high and low performers was Self-Reliance for the population sample studied. Although this might justify the exclusion of this particular scale in the MMP3™, it was decided, at least temporarily, to retain it based numerous studies that support the importance of self-reliance as a significant contributor to performance (Bar-On et al., 2006).

While the results in Table 4 indicate that nearly all of the MMP3™ scales are capable of significantly distinguishing between high and low performers, the findings in Table 5 suggest that all of the scales, including Self-Reliance, are capable of predicting performance. These findings would appear to justify retaining the Self-Reliance scale in the questionnaire. Moreover, the overall multivariate correlation—i.e., the Multiple R—between the MMP3™ scales and self-reported performance is 0.56 (*F* = 7.39, *p* < 0.001) suggesting that it possesses relatively high predictive validity; and this was expected from the scales' relatively high reliability as was previously mentioned. The three most robust predictors of performance appear to be situational awareness (β = 0.274, *t* = 4.60, *p* < 0.001), coping and endurance (β = 0.154, *t* = 3.01, *p* = 0.003), and engagement (β = 0.115, *t* = 3.24, *p* = 0.001).

In a recently approved doctoral dissertation (Conroy, 2017), Dr. Richard Conroy demonstrated that the factors assessed by the MMP3™ adequately predict effective leadership. More succinctly, this instrument was shown to be a robust predictor of “transformational leadership” in a sample of 454 senior law enforcement officers. The predictive model that emerged, from Multiple Regression Analysis, indicated that most of the variance of this type of leadership can be significantly accounted for [R = 0.76 (*F* = 35.00, *p* < 0.001)] by the MMP3™. A re-examination of the dataset suggests that its predictive ability is even greater than was originally thought.

## Discussion

### The key findings

The key findings presented in this article suggest that the MMP3™ is a reliable and valid measure of performance including leadership. Moreover, this psychometric instrument has been methodically developed based on (1) a systematic search of the literature, (2) input from expert consultants who have worked in various aspects of human performance as well as (3) the application of multivariate statistics designed to examine its factorial structure, reliability and validity. Furthermore, the MMP3™ addresses the need for developing more reliable and valid multifactor measures of performance in pre-employment testing. In addition to comprehensively assessing the potential for performance in the workplace, it is hoped that it can be applied elsewhere as is discussed below. The findings also suggest that three of the most robust predictors of occupational performance appear to be (1) possessing situational awareness and being attentive to detail, (2) being able to effectively cope with stress as well as (3) being totally engaged that significantly impacts one's motivational level. According to how these factors are described and assessed by the MMP3™, this means that performance is driven by being attentive to one's immediate surroundings, paying attention to detail and not getting distracted. Additionally, effective performance also requires the ability to cope well with stress and function well under pressure. Last, high performers need to be engaged in their work and feel passionate about what they do in order to be sufficiently motivated to function at their best.

### The importance of the findings

One of the most important findings revealed in this article is that the MMP3™ is capable of concomitantly assessing five different groups of significant contributors to performance, comprising a total of 22 factors, including the following: (1) two physical factors; (2) eight cognitive factors; (3) four intra-personal factors; (4) three inter-personal factors; and (5) five motivational factors. In addition to confirming more that 80% of the ideas presented in the author's previous publication moreover (Bar-On, 2016), the results presented here empirically support the importance of physical (Boles et al., 2004; Fritz and Sonnentag, 2005; Meerding et al., 2005; Conn et al., 2009; Pronk and Kottke, 2009), cognitive (Motowidlo et al., 1986; Mento et al., 1987; Pearson, 1987; Janssen and Van Yperen, 2004; Cote and Miners, 2006; Hill and Lewicki, 2006), intra-personal (Motowidlo et al., 1986; Matteson and Ivancevich, 1987; Sullivan and Bhagat, 1992; Kuncel et al., 2004; Bar-On et al., 2006; Martinuzzi, 2009), inter-personal (Babin and Boles, 1996; Schwepker and Ingram, 1996; Van Scotter and Motowidlo, 1996; Janssen and Van Yperen, 2004; Lennick and Kiel, 2007; Baker and O'Malley, 2008; Hsu, 2008), and motivational (Weitz et al., 1986; Cotton, 1993; Becker et al., 1996; Diefendorff et al., 2002; Locke and Latham, 2002; Halbesleben and Wheeler, 2008; Markos and Sridevi, 2010; Rich et al., 2010) predictors of performance proposed by others; and this, in turn, appears to help validate what the MMP3™ assesses as well. This also empirically confirms the value of combining the above-mentioned groups of contributors to better predict performance, which justifies the importance of developing a multifactor conceptual and psychometric model that is designed to comprehensively evaluate the *whole person* when attempting to study, assess and enhance human behavior and performance.

The novelty of the MMP3™, as well as this publication in the professional literature, is that the above-mentioned five different groups of contributors to performance can be assessed by employing only *one* psychometric instrument, thus reducing the need to combine various different instruments to evaluate all of these key factors; and this, in turn, is expected to reduce the time and cost involved in psychological testing.

In addition to its assessment component, it is important to emphasize that the MMP3™ has a development component that will automatically provide a number of suggestions for strengthening the individuals' weakest contributors to performance indicated by their lowest scores. Thus, the MMP3™ can be contextualized as a comprehensive operational framework designed to help understand why some people perform better than others and to determine which contributing factors need to be strengthened in order to enhance performance in individuals who are underperforming.

Depending upon the outcome of future predictive validity and incremental validity studies, it is possible that the MMP3™ will compare favorably with other psychometric instruments used to predict performance in the workplace and elsewhere. This is, cautiously, based on what has been presented here compared with findings from other publications describing the predictive validity of most pre-employment testing involving cognitive factors and personality traits for example. More specifically, the present article revealed a predictive coefficient of 0.56 suggesting that the MMP3™ scales are capable of assessing more than 30% of the variance that explains occupational performance accounting for nearly a third of the factors that predict performance in the workplace which is significant in the field of test-construction. These findings suggest that the MMP3™ could be a potentially valuable and desirable tool when this is compared with the results of very large meta-analyses that have been conducted on the predictive ability of cognitive and personality tests. More succinctly, Morgeson and his colleagues reported findings from 13 meta-analyses (*n* = 40,230) indicating that the average predictive coefficient of cognitive testing is 0.25 accounting for only 6% of the variance of occupational performance (Morgeson et al., 2007, p. 700); and based on 12 meta-analyses (*n* = 23,413), the average predictive coefficient of personality traits is only 0.15 accounting for a mere 2% of the variance (Morgeson et al., 2007, p. 705).

The importance of the MMP3™, as an assessment, development and research instrument, will depend on the extent to which it will be applied and the degree to which it will help improve human performance. If it can be effectively applied in one or more of the following areas and prove to be useful moreover, it is reasonable to assume that it will demonstrate both importance and value as an operational model of performance: (1) parenting; (2) education; (3) human resources; (4) healthcare; and (5) research designed to study and improve performance. These potential applications were described, in detail, by the author in his first publication on the Multifactor Measure of Performance™ (Bar-On, 2016, p. 113–115). An additional application of this psychometric instrument is currently being examined for the purpose of assessing and training emergency responders and managers, aimed at enhancing their performance in natural and man-made disasters. The author is currently working with Professor Isaac Ashkenazi, an internationally acknowledged expert in this field, to develop a customized version of the MMP™ which will include a VR (Virtual Reality) application designed to facilitate the assessment and development of emergency managers and crisis leaders.

If one takes into account the above-mentioned features, psychometric strengths and potential applications of the MMP3™, these would be the primary reasons for practitioners and researchers to apply this assessment and development instrument. To verify the potential of the MMP3™ however, additional studies will need to be conducted on larger and more diverse population samples aimed at examining its discriminatory, predictive and incremental validity. Future studies will also need to address the potential limitations of the present study that are discussed below.

### Limitations of the present research and the need for future studies

One of the basic approaches used in developing the Multifactor Measure of Performance™ also represents one of its potential limitations. More specifically, the contributors to performance that this questionnaire was designed to measure were those that were reviewed in the literature by the author who selected those he thought to be the key contributors to performance. It is reasonable to assume that other researchers would have reviewed the literature differently, possibly would have selected other predictors and might have also defined them definitely. Additionally, others might have decided to combine what were perceived to be similar factors and divide other factors into two or more separate factors. It is therefore important to receive additional input on the author's approach to developing this particular model.

Another potential limitation of the present study was not including more experts in the field, who could have provided additional ideas regarding important contributors to and predictors of performance, their description of these factors and selection of items designed to assess them.

It is also important to note that additional MMP3™ data are currently being collected by the author, colleagues and other researchers; and results from these and future studies might or might not confirm this questionnaire's estimated factorial structure, psychometric properties, and strengths as presented in this article.

To reiterate, the MMP3™ will need to be examined on larger and more diverse population samples across cultures. In order to receive a clearer and more accurate picture of the MMP3™'s factorial structure, CFA will be conducted *after* the author has obtained data from significantly larger samples. In addition to more extensively examining factorial structure and validity, the MMP3™'s discriminant, predictive and, especially, incremental validity studies will also need to be conducted as was previously mentioned.

An additional limitation of the current study can be seen in the way in which the MMP3™'s predictive validity has been examined. While the approach applied to estimate occupational performance, described in the data collection section, provided an *approximation* of how the subjects might be performing, it therefore has limitations and will eventually require the use of more objective methods in future studies such as (1) actual performance ratings completed by supervisors and co-workers as well as (2) comparing criterion groups of identified high and low performers for significant differences. Furthermore, future research will need to examine what types of human behavior and performance the MMP3™ predicts and how well. In addition to general occupational performance, its ability to predict and improve teamwork and leadership will be studied as well. Additionally, its ability to evaluate and enhance parenting, academic performance as well as physical and psychological health will also need to be researched. Test-retest reliability studies will also need to be conducted in addition to MMP3™'s internal consistency reliability which was examined in the present article.

In that the norming and validation of psychometric instruments is a very lengthy process, it will take time before MMP3™'s exact structure, reliability and validity can be more fully understood; and the author welcomes researchers, students, and practitioners to apply and examine the MMP3™ in future studies to help facilitate its continued norming, validation and application. The author plans to have the questionnaire translated from English to a number of different languages, which will both facilitate its continued norming and validation in order to continue studying the key contributors to performance as well as potentially expand its applicability.

## Author contributions

RB-O is the author of this paper, which is his “Inaugural Article” as an Associate Editor in the Organizational Psychology Section of Frontiers in Psychology. His professional background and interest focuses on the study, development and application of multifactor models and measures of performance. Since 1978, he has developed 12 different psychometric instruments designed to assess and develop various aspects of human performance including the Emotional Quotient Inventory™ (EQ-i™). This article describes the Multifactor Measure of Performance™ (MMP™), which was developed to comprehensively measure and enhance what RB-O identified as key contributors to human performance. The MMP™, the concept it was designed to measure and the research involved in developing it are described in detail.

### Conflict of interest statement

RB-O is the founder of Bar-On Test Developers LLC and currently directs the R&D at Bar-On Test Developers.

## Footnotes

^2^Statistica 12.7 is referenced in https://en.wikipedia.org/wiki/Statistica.

^3^“Sub-factors” describe multiple smaller factors, two to six in the present study, that load on the same factor, as are observed in factors 3, 15, and 20 in Tables 1A,C. This term is descriptive in nature rather than statistical.

^4^“Sub-scales” describe smaller scales clustered within larger ones. In the MMP3™, they appear in the following scales: Key Cognitive Competencies, Finding Meaning and Acting Responsibly, and Motivational Drive. Once again, it is important to note that this term is descriptive rather than statistical.

^5^A “composite scale” refers to a scale that comprises sub-scales (Key Cognitive Competencies, Finding Meaning and Acting Responsibly, and Motivational Drive); and a composite scale score is calculated by averaging its sub-scale scores. It is important to note that this is a descriptive rather than a statistical term.

## Appendix

**Appendix A:** The eight categories listed below describe what pre-employment tests, in the 20th edition of the *Mental Measurement Yearbook*, appear to evaluate based on a random sample of 120 of the 359 tests that were examined.

1. Vocational & career interests [11 (9.2%)]
2. Employability & general / specific employment skills needed in various occupations [24 (20.0%)]
3. Cognitive intelligence & academic ability / skills / readiness / achievement [44 (36.7%)]
4. Intra-personal competencies & personality traits [17 (14.2%)]
5. Inter-personal compatibility & communication skills [6 (5.0%)]
6. Managerial & leadership skills [4 (3.3%)]
7. Responsibility / commitment / ethics / integrity / honesty / dependability [10 (8.3%)]
8. Potential for disruptive psychological problems and/or criminal behavior [4 (3.3%)]

*The number of tests found in each category are included in brackets following each of the eight categories*.

**Appendix B:** A comparison of the 26 MMP1™ scales below with those retained in the MMP3™ [in brackets] indicates that 85% of the original scales were retained based on conducting nine sets Exploratory Factor Analysis.

1. Physical Fitness [*retained in the MMP3*™]
2. Discomfort Tolerance [*retained in the MMP3*™]
3. General Cognitive Competence [*retained in the MMP3*™]
4. Situational Awareness [*retained in the MMP3*™]
5. Applying Experience [*retained in the MMP3*™]
6. Flexibility [*retained in the MMP3*™]
7. Resourcefulness [*retained in the MMP3*™]
8. Decision-Making [*retained in the MMP3*™]
9. Action-Planning
10. Rapid Implementation
11. Self-Awareness
12. Self-Control [*retained in the MMP3*™]
13. General Coping Ability [*retained in the MMP3*™]
14. Self-Reliance [*retained in the MMP3*™]
15. Decisiveness [*retained in the MMP3*™]
16. Courage [*retained in the MMP3*™]
17. Meaningfulness [*retained in the MMP3*™]
18. Self-Motivation [*retained in the MMP3*™]
19. Engagement [*retained in the MMP3*™]
20. Determination [*retained in the MMP3*™]
21. Perseverance [*retained in the MMP3*™]
22. Humility
23. Social-Awareness [*retained in the MMP3*™]
24. Social Responsibility [*retained in the MMP3*™]
25. Connectedness [*retained in the MMP3*™]
26. Well-Being [*partly retained in the MMP3*™]
